# Effect of ****α****-Lipoic Acid on Oxidative Stress in End-Stage Renal Disease Patients Receiving Intravenous Iron

**DOI:** 10.1155/2014/634515

**Published:** 2014-03-05

**Authors:** Arif Showkat, William R. Bastnagel, Joanna Q. Hudson

**Affiliations:** ^1^Division of Nephrology, Department of Medicine, University of Tennessee Health Science Center, 956 Court Avenue, Suite B224, Memphis, TN 38163, USA; ^2^Emergency Department, Methodist Affiliated Hospitals, Memphis, TN 38163, USA; ^3^Department of Clinical Pharmacy, University of Tennessee Health Science Center, Memphis, TN 38163, USA

## Abstract

Oxidative stress is associated with increased risk of cardiovascular disease in end-stage renal disease (ESRD) patients. Intravenous (IV) iron has been shown to increase oxidative stress. The aim of the study was to evaluate changes in oxidative stress markers following administration of IV sodium ferric gluconate (SFG) to ESRD patients with and without administration of the antioxidant, **α**-lipoic acid. This is an open-label, crossover study. 125 mg of IV SFG was administered during control (C) and intervention (I) visits. During the I visit, 600 mg of **α**-lipoic acid was given orally prior to IV SFG. Blood samples were collected at defined time periods for F_2_-isoprostane (FIP), lipid hydroperoxide (LHP), malondialdehyde (MDA), and iron indices. We recruited ten African-American ESRD subjects: 50% male; mean age 45 ± 9 years; mean hemoglobin 13 ± 1 g/dL; ferritin 449 ± 145 ng/mL; transferrin saturation 27 ± 4%. There were no significant differences in iron indices between the two visits after IV SFG. MDA, FIP, and LHP increased significantly for both C and I visits with a greater increase in the I group. Administration of IV SFG results in an acute rise in oxidative stress in ESRD patients. In contrast to previous studies, administration of **α**-lipoic acid was associated with a greater increase in oxidative stress.

## 1. Introduction

Patients with end-stage renal disease (ESRD) on hemodialysis (HD) have very high cardiovascular mortality [1]. Interestingly, the usual risk factors for atherosclerotic diseases in the general population fail to explain the increased cardiovascular mortality in the HD population. For example, unlike the general population, mild-to-moderate elevation of cholesterol or blood pressure does not correlate with increased atherosclerotic complications or mortality in HD patients. As a result, several nontraditional uremia-related risk factors have been considered, including elevated levels of oxidative stress [1, 2].

Intravenous (IV) iron administration has become an integral part of anemia management in ESRD patients; however, exposure to IV iron is among the factors associated with the increase in oxidative stress in the dialysis patients [3–6]. Whether chronic exposure to low maintenance doses of IV iron increases the risk of cardiovascular events has yet to be determined.

The oxidative stress markers, malondialdehyde (MDA), lipid hydroperoxide (LHP), and F_2_ isoprostane (FIP), represent products formed by the reactive oxygen species generated following administration of IV iron. MDA is an end product of peroxidation of polyunsaturated fatty acids and is a reactive aldehyde that causes toxic stress in cells [7]. MDA has been shown to increase rapidly (within 15 to 30 minutes) in patients with chronic kidney disease (CKD) and in HD patients following IV iron administration [3, 4]. Formation of LHP is indicative of lipid peroxidation, which results in oxidative damage in cell membranes, lipoproteins, and other lipid-containing structures [8]. Isoprostanes are prostaglandin-like substances produced in vivo primarily by free radical-induced peroxidation of arachidonic acid [9]. FIPs are a group of 64 compounds isomeric in structure to cyclooxygenase-derived PGF2 and serve as ideal markers of oxidative stress since they are chemically stable, increase substantially during oxidant injury, and are specific products of peroxidation.

Interventions that have been attempted to counteract the effects of heightened oxidative stress in dialysis patients include administration of substances with antioxidant activities, such as *α*-tocopherol [10], angiotensin converting enzyme inhibitors [11], and vitamin E [5], a hemolipodialysis procedure [12], and the use of a vitamin E coated dialyzer membrane [13]. Administration of N-acetylcysteine prior to IV iron administration has also been reported to reduce oxidative stress in patients with CKD; however, the results have been equivocal [3, 6].

The antioxidant *α*-lipoic acid is more palatable than N-acetylcysteine and has shown benefit in preventing events associated with oxidative stress including diabetic neuropathy and glomerular injury [14]. In humans, *α*-lipoic acid has been used successfully to treat symptomatic diabetic polyneuropathy, an effect believed to be induced by enhanced formation of reactive oxygen species [14]. In a comparative study involving *α*-lipoic acid and *α*-tocopherol supplementation in human subjects, *α*-lipoic acid administration was associated with comparable decreases in oxidative stress markers (urinary FIP, plasma protein carbonyls, and LDL oxidizability); thus, it is plausible that administration of this agent may reduce oxidative stress induced by IV iron [15].

The purpose of this study was to test the hypothesis that a single oral dose of *α*-lipoic acid given prior to administration of IV sodium ferric gluconate attenuates formation of oxidative stress markers and to determine if changes in iron indices correlate with changes in these markers.

## 2. Materials and Methods

### 2.1. Study Subjects

Adult male and female subjects with ESRD requiring chronic HD and under the care of nephrologists at the University of Tennessee, Division of Nephrology, were evaluated for participation in this study. Eligible subjects had to be between 18 and 80 years of age, receiving HD 3 times a week for at least 3 months, without residual kidney function (i.e., no urine output). Subjects were excluded if they had any form of dialysis catheter, had an active infection, had a documented history of chronic liver disease, elevated iron indices indicating iron overload (i.e., serum ferritin > 800 ng/mL and/or transferrin saturation >50%), were pregnant or breastfeeding, or were taking medications with antioxidant properties including HMG-CoA reductase inhibitors, angiotensin converting enzyme inhibitors, vitamin E, aspirin, or any form of steroids. Previous studies reported a 50% change in the oxidative stress marker MDA following administration of IV iron; therefore, the target sample size was 10 subjects to allow detection of a 25% change in oxidative stress markers with a power of 0.87 (*α* = 0.05) [3]. The institutional review board of the University of Tennessee Health Sciences Center and the University of Tennessee Clinical Research Center (UT CRC) approved the study. All study procedures were conducted in the UT CRC facilities. Informed written consent was obtained from all participants.

### 2.2. Study Design

This was a single center, prospective, controlled study. Eligible subjects participated according to the study protocol, which required two separate visits, intervention and control visits. The sequence of control and intervention visits was assigned randomly for each participant (i.e., intervention followed by control visit or control visit followed by intervention visit). The study visits were scheduled on nondialysis days and were at least one week apart. Subjects were required to fast overnight before each visit. During both the control and intervention visits subjects received 125 mg of sodium ferric gluconate (Ferrlecit, Watson Pharmaceutical Inc.) administered intravenously over 10 minutes. Blood samples were drawn for measurement of the oxidative stress markers and iron indices at the start (time 0) and 15, 30, 60, 90, 120, 150, and 180 minutes after administration of IV iron. The oxidative stress markers measured were MDA, LHP, and FIP. Iron indices included serum iron, total iron binding capacity (TIBC), transferrin saturation [(serum iron/TIBC) × 100], and serum ferritin. Hemoglobin, hematocrit, and serum albumin were measured from the sample drawn at time 0. Blood pressure and heart rate were monitored prior to the infusion and 30 and 60 minutes following the infusion. During the intervention visit participants received 600 mg of racemic *α*-lipoic acid (Nutraceutical Sciences Institute, Boynton Beach, FL) orally 30 minutes prior to IV iron administration. During the intervention visit, blood glucose level was measured at the time of *α*-lipoic acid administration and at 60 minutes after IV iron administration.

The 600 mg dose of *α*-lipoic acid has been used successfully to treat polyneuropathy in diabetic patients [14, 16]. Information on the pharmacokinetics of a single dose of 600 mg in patients with ESRD indicates that this agent is rapidly absorbed (*t*_max⁡_ 30 minutes) with a half-life of approximately 21 minutes and that urinary elimination is not a significant pathway of elimination suggesting that the dosing regimen selected for this study is appropriate [17, 18]. Based on this information we chose to give the 600 mg dose of *α*-lipoic acid 30 minutes prior to the administration of IV iron.

### 2.3. Laboratory Analysis

Blood samples from each time point were collected in heparinized vacutainers. The plasma was separated by centrifugation at 1500 g for 15 minutes and stored at −70°C until assayed. Plasma MDA concentrations were measured using the thiobarbituric acid methodology; plasma LHP concentrations were determined by a colorimetric assay after methanol-chloroform extraction, and FIP was measured using enzyme immunoassay methodology [19, 20]. Reagents were obtained from Cayman Chemical Co., Ann Arbor, MI. The local UT CRC laboratory measured all iron indices, albumin, hemoglobin, and hematocrit.

### 2.4. Statistical Analysis

Continuous data was presented as mean ± SD or as median (interquartile range). Repeated measures analysis of variance was used to test the trend in serum iron, total iron binding capacity, transferrin saturation, and ferritin over time. A mixed-effect model for repeated measures was used to determine the effect of the *α*-lipoic acid administration on the oxidative stress markers. For each oxidative stress marker model, respective baseline oxidative stress marker data have been introduced as a covariate. Post hoc tests were performed by using contrast analysis to test differences between control and intervention at each time point and also between baseline to 15, 30, 60, 90, 120, 150, and 180 minutes follow-up. Differences in the area under the curve (AUC) for the oxidative stress markers were compared using a paired *t*-test. All tests were 2-sided and a *P* value < 0.05 was considered significant, unless otherwise stated. Statistical analysis was conducted using SAS version 9.2 (SAS Institute Inc., Cary, NC, USA).

## 3. Results

### 3.1. Study Subjects

Ten African-American ESRD subjects met inclusion criteria and were enrolled in the study. Five were male, 9 had hypertension, and 4 had diabetes. The average age was 44.9 ± 9.1 years. All patients were dialyzed three times a week using a high-flux biocompatible membrane according to a standardized treatment protocol for outpatient chronic HD patients. Nine subjects were receiving scheduled IV iron as iron sucrose; eight subjects received therapy 3 times a week and 1 subject received weekly doses. The average iron sucrose dose was 42.5 ± 26.5 mg per dialysis session. Seven patients were on erythropoietic stimulating agents (ESAs) administered with every dialysis session. The average erythropoietin dose was 3050 ± 3009 units per dialysis session.

### 3.2. Iron Indices

**Figure 1:**
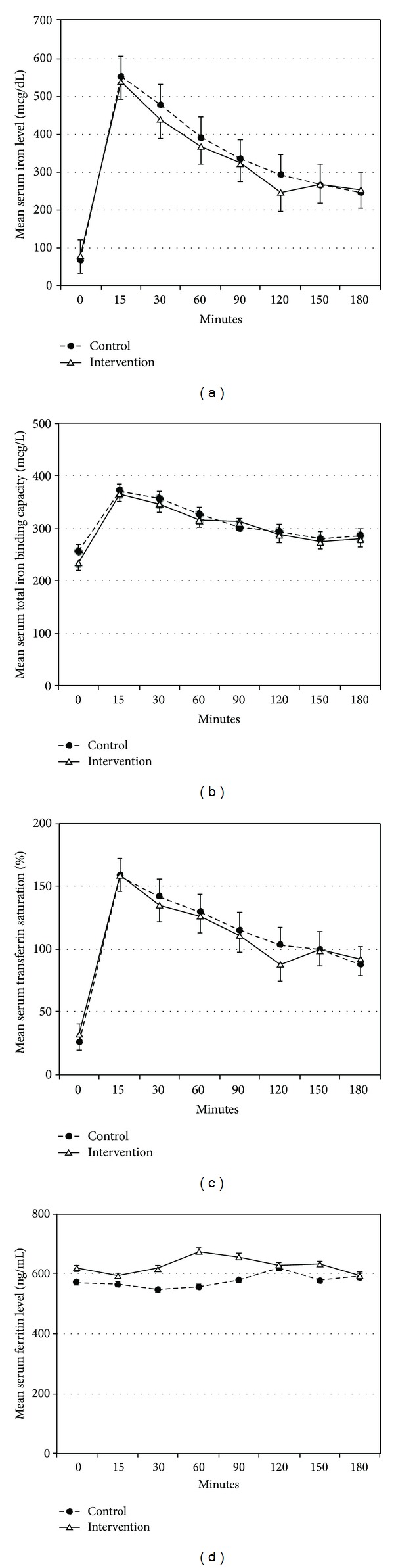
Changes in serum iron (a), total iron binding capacity (b), transferrin (c), and ferritin (d) levels (mean ± SD) over time after intravenous iron infusion in control and intervention groups.

There were no significant differences in laboratory values at baseline between the control and intervention visits except for the transferrin saturation, which was higher for the intervention visit (Table 1). Changes in iron indices after infusion of IV iron are shown in Figures 1(a), 1(b), 1(c), and 1(d). There was a sharp increase in serum iron, total iron binding capacity, and transferrin saturation levels after iron infusion that reached a peak level within 15 minutes. There was no significant change in serum ferritin after IV iron infusion during the sampling period. The changes in iron indices over time after iron infusion were similar for the control and intervention visits.

### 3.3. Oxidative Stress Markers

**Figure 2:**
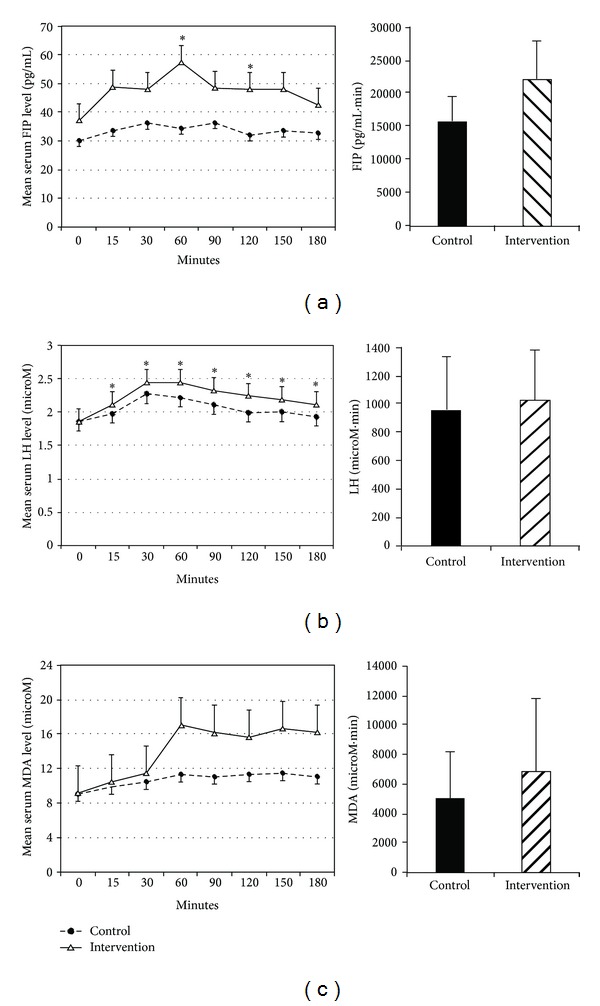
Changes in serum F_2_-isoprostane (FIP) (a), lipid hydroperoxide (LHP) (b), and malondialdehyde (MDA) levels (mean ± SD) over time after intravenous iron infusion in control and intervention groups. In mixed-effect model with time as repeated measure a significant effect of *α*-lipoic acid (*P* < 0.001), time (*P* < 0.001), and *α*-lipoic-by-time interaction (*P* < 0.01) was observed for changes in serum FIP [TOEP(2) matrix] and LHP level (TOEP matrix) and a significant effect of time (*P* < 0.001) was observed for changes in serum MDA level [ANTE(1) matrix]. *Significant differences between groups, *P* < 0.05. The inset depicts the area under the curve (AUC) of oxidative stress marker levels FIP (*P* = 0.0045), LHP (*P* < 0.001), and MDA (*P* = 0.1087) in control and intervention groups.

Baseline serum FIP, LHP, and MDA levels were similar between the control and intervention visits (Table 1). Mean serum levels of all 3 oxidative stress markers increased significantly after administration of sodium ferric gluconate and reached peak levels at 60 minutes (Figure 2). There was no significant correlation between the oxidative stress marker levels at baseline and the variables of age, gender, baseline hemoglobin, serum iron, ferritin, total iron binding capacity, transferrin saturation, ESAs, or IV iron dose.

There were differences in serum FIP levels after *α*-lipoic acid administration characterized by a significant global effect of *α*-lipoic acid (*P* = 0.0017), time (*P* < 0.0001), and *α*-lipoic × time interaction (*P* = 0.0187) (Figure 2(a)). In particular, the serum FIP level was significantly higher after *α*-lipoic acid administration at 60 and 120 minutes. Similarly, serum LHP levels showed a significant global effect of *α*-lipoic acid (*P* < 0.0001), time (*P* < 0.0001), and *α*-lipoic × time interaction (*P* = 0.0165) (Figure 2(b)). Compared to the control group, the serum LHP level increased significantly in the intervention group after *α*-lipoic administration at 15 minutes and remained elevated throughout the study period. There was no overall effect of *α*-lipoic acid (*P* = 0.1091) or any interaction between *α*-lipoic acid and time (*P* = 0.4037) on serum MDA level (Figure 2(c)). However, a significant time effect (*P* < 0.0001) was observed on serum MDA level indicating that after IV iron infusion the serum MDA level changed from baseline, but there was no significant difference between the control and intervention groups.

The area under the plasma concentration versus time curves (AUC) for each of the oxidative stress markers is shown for the intervention and control groups in Figures 2(a), 2(b), and 2(c). The AUC for intervention visits is significantly greater compared to control visits for FIP (22046 ± 5921 versus 15694 ± 3873 pg/mL × min, *P* = 0.0045) and LHP (1025 ± 353 versus 956 ± 375 *μ*M × min⁡, *P* < 0.001) indicating greater production of these oxidative stress markers following *α*-lipoic acid administration. There was no significant difference in the AUC between the intervention and control visits for MDA (6851 ± 5020 versus 5043 ± 3134 *μ*M × min, *P* = 0.1087).

## 4. Discussion

This study demonstrated that IV iron administration in hemodialysis patients was associated with a rise in oxidative stress markers; however, pretreatment with the antioxidant *α*-lipoic acid failed to prevent this increase in oxidative stress. In fact, *α*-lipoic acid administration was associated with a further rise in oxidative stress markers demonstrating a potential prooxidant effect.

IV iron and ESAs administration have become an integral part in the management of anemia in hemodialysis patients. In recent years, the safety concerns of ESAs have led to the significant restrictions on the use of ESAs and renowned interest in the more aggressive use of IV iron [21]. The association of IV iron administration and increases in oxidative stress markers in CKD and dialysis patients is well documented [3–6]. Some variations in oxidative stress response are reported in hemodialysis patients among the available iron preparations (sodium ferric gluconate, iron dextran, and iron sucrose) with sodium ferric gluconate being associated with the highest serum MDA levels compared to other iron preparations [22].

Iron as a metal has redox activity. It can accept an electron in low valence state Fe^2+^ (ferrous) and donate an electron in high valence state Fe^3+^ (ferric) [23]. Both the storage form of iron as ferritin and the carrier form as transferrin keep iron in a stable state and prevent participation of unbound iron in redox activities. In disease conditions where there are possibilities for superoxide (O_2_^−^) production, the storage form of iron, ferritin becomes vulnerable to the attack by O_2_^−^. This results in release of stored metal iron in Fe^2+^ state [O_2_^−^ + Fe^3+^ → O_2_ + Fe^2+^]. The released iron can react with H_2_O_2_ and produce OH radicals [H_2_O_2_ + Fe^2+^ → OH^•^ + OH^−^ + Fe^3+^]. The OH^•^ is an extremely powerful reactive oxygen species (ROS). This ROS can attack any class of biological macromolecules and cause various damaging reactions such as inactivation of enzymes, depolymerization of polysaccharides, and lipid, protein, and carbohydrate peroxidation. Peroxidation of lipid, protein, and carbohydrate by the ROS produces different macromolecules. These macromolecules have a longer half-life than the ROS itself and can be measured. They represent the markers for oxidative stress.

In the current study oversaturation of iron (transferrin saturation >100%) was observed within 15 minutes of IV iron administration (Figure 1). This indicates the availability of free iron, which becomes susceptible to the action of superoxide and formation of ROS. The rise in transferrin saturation is preceded by the upsurge in oxidative stress marker levels. The increase in oxidative stress markers in intervention group cannot be attributed to increases in iron indices since the change in iron indices was similar with and without *α*-lipoic acid administration (Figure 1). Evidence of the prooxidant effect of *α*-lipoic acid comes from observations of a significant effect of *α*-lipoic acid on FIP and LHP concentrations and the higher AUC observed for FIP and LHP following *α*-lipoic acid administration (Figure 2). The prooxidant effect of *α*-lipoic acid has been reported in several in vitro and animal studies, but not in humans [24, 25]. Our study suggests such an effect in humans also.

The unexpected findings from this study may be attributed to the actions of dihydrolipoic acid (DHLA), the reduced thiol form of *α*-lipoic acid. *α*-lipoic acid is normally reduced to DHLA after cellular intake. Both *α*-lipoic acid and DHLA are powerful antioxidants and produce antioxidant actions by chelation of several metals including iron [26, 27]. In contrast, DHLA can also produce a prooxidant effect in the presence of a transitional metal such as iron by reducing Fe^3+^ to Fe^2+^, which can promote oxidative damage by hydroxyl radicals. DHLA can also generate sulfur-containing radicals, which can damage certain proteins, such as *α*-1 antiproteinase and creatine kinase [28].

The prooxidant effect of antioxidant substances is not unique to *α*-lipoic acid. Vitamin C and N-acetylcysteine usually have well-established antioxidant effects; however, in the presence of inflammation and free iron, administration of these substances has been associated with increase in oxidative stress marker levels [29].

In conclusion, this study highlights the potential undesirable interaction of antioxidant therapy with *α*-lipoic acid and available free iron in hemodialysis patients. Supplementation of *α*-lipoic acid in such conditions does not appear to be beneficial or innocuous and may further exacerbate oxidative stress injury. The effect of chronic supplementation of *α*-lipoic acid in patients with elevated free iron and inflammation warrants further study.

## Figures and Tables

**Table 1:** Baseline values for control and intervention visits.

Variable	Control visit	Intervention visit	*P* value
Hemoglobin (g/dL)	12.85 (11.6, 14)	13 (11.7, 13.7)	0.675
Hematocrit (%)	38.94 ± 3.68	39.57 ± 3.42	0.718
Serum albumin (g/dL)	3.79 ± 0.27	3.86 ± 0.34	0.679
Serum iron (*µ*g/dL)	68 ± 12	79 ± 23	0.170
Total iron binding capacity (*µ*g/dL)	256 ± 49	234 ± 38	0.280
Transferrin saturation (%)	27 (24, 28)	31 (30, 36)	0.010
Serum ferritin (ng/mL)	570 ± 241	617 ± 397	0.880
Malondialdehyde (*µ*mol/L)	9.04 ± 5.29	9.12 ± 5.16	0.973
Lipid hydroperoxide (pg/mL)	1.86 ± 0.78	1.86 ± 0.73	0.943
F_2_ isoprostanes (*µ*mol/L)	37.07 ± 13.28	30.04 ± 6.89	0.161

Values are expressed as mean ± SD or median (interquartile range). Wilcoxon signed-rank test was used for hemoglobin and transferrin saturation; paired *t*-test was used for all other variables.
